# Divergence of the Yeast Transcription Factor *FZF1* Affects Sulfite Resistance

**DOI:** 10.1371/journal.pgen.1002763

**Published:** 2012-06-14

**Authors:** Elizabeth K. Engle, Justin C. Fay

**Affiliations:** 1 Molecular Genetics and Genomics Program, Washington University, St. Louis, Missouri, United States of America; 2 Department of Genetics and Center for Genome Sciences and Systems Biology, Washington University School of Medicine, St. Louis, Missouri, United States of America; Fred Hutchinson Cancer Research Center, United States of America

## Abstract

Changes in gene expression are commonly observed during evolution. However, the phenotypic consequences of expression divergence are frequently unknown and difficult to measure. Transcriptional regulators provide a mechanism by which phenotypic divergence can occur through multiple, coordinated changes in gene expression during development or in response to environmental changes. Yet, some changes in transcriptional regulators may be constrained by their pleiotropic effects on gene expression. Here, we use a genome-wide screen for promoters that are likely to have diverged in function and identify a yeast transcription factor, *FZF1*, that has evolved substantial differences in its ability to confer resistance to sulfites. Chimeric alleles from four *Saccharomyces* species show that divergence in *FZF1* activity is due to changes in both its coding and upstream noncoding sequence. Between the two closest species, noncoding changes affect the expression of *FZF1*, whereas coding changes affect the expression of *SSU1*, a sulfite efflux pump activated by *FZF1*. Both coding and noncoding changes also affect the expression of many other genes. Our results show how divergence in the coding and promoter region of a transcription factor alters the response to an environmental stress.

## Author Summary

Changes in gene regulation are thought to play an important role in evolution. While variation in gene expression between species is common, it is hard to identify the phenotypic consequences of this variation since many changes in gene expression may have subtle or no phenotypic effects. In this study, we investigate changes in sulfite resistance and gene expression caused by the transcription factor, *FZF1*, that has evolved rapidly during the divergence of related yeast species. We find that divergence in the ability of *FZF1* to confer sulfite resistance is mediated by changes in its expression as well as changes in its protein structure, both of which cause changes in the expression of other genes. Our results show how the combination of multiple changes within a transcription factor can produce substantial changes in phenotype and the expression of many genes.

## Introduction

Transcriptional regulation plays a key role in development and an organism's response to physiological and environmental changes. However, changes in gene regulation that occur over the course of evolution are more difficult to interpret. Genome-wide patterns of gene expression divergence show that while many aspects of regulation are conserved between distantly related species [1]–[3], there is also extensive variation in gene expression levels within and between closely related species [4]. In many, but not all instances, gene expression divergence is consistent with a neutral model of evolutionary change [5]–[8]. Yet, understanding regulatory divergence requires identifying the genetic basis of divergence in gene expression and knowing which changes in gene expression translate into changes in phenotype and fitness.

Substantial progress has been made in understanding the genetic basis of regulatory divergence. Changes in gene expression are influenced by both *cis*-regulatory sequences and *trans*-acting factors, with *cis*-regulatory changes being enriched in interspecific comparisons [9], [10]. Expression changes caused by *cis*-regulatory elements frequently involve gain or loss of transcription factor binding sites, e.g. [11], [12], although other changes, such as nucleosome position, can also play an important role [13]. Even when changes in gene expression can be attributed to specific *cis*-regulatory elements, the phenotypic consequences of such changes are hard to know, especially if they depend on the combined effects of many *cis*-regulatory changes. While changes in *trans*-acting factors can simultaneously influence the expression of many genes, significant efforts are needed to identify the genetic basis of *trans*-acting changes in gene expression.

The phenotypic effects of changes in gene expression have in some cases been identified [14]. This has primarily been accomplished by mapping, association and transgenic studies that identify genetic changes underlying a phenotype. While these approaches typically identify changes in protein coding sequences, *cis*-regulatory changes are more frequently found to underlie interspecific compared to intraspecific differences [14]. Furthermore, changes in protein coding sequences can affect the expression of many genes [15], [16], and in some cases their phenotypic effects depend on multiple differentially expressed genes [17].

What has been more difficult to investigate is the combined influence of multiple regulatory changes. Multiple changes of small effect may frequently go undetected, at least individually, but together could have a substantial impact on divergence [18]. Evidence for adaptive evolution via multiple *cis*-regulatory changes has been found based on concerted changes in the expression of genes that function in the same pathway or biological process [19]–[21]. Multiple *cis*-regulatory changes at a single locus have also been found to make substantial contributions to phenotypic divergence between species [22]–[26].

Statistical tests of neutrality are particularly well-suited to identifying multiple adaptive substitutions at a single locus since multiple substitutions are often needed to detect a significant deviation from a neutral pattern of molecular evolution. Rapidly evolving noncoding sequences have been identified in a number of species [27]–[30], and in some instances are known to cause notable changes in gene expression [31], [32]. Although tests of neutrality rely on the concentration of multiple changes at single loci, clustering of changes may occur if there are genetic, developmental or selective constraints at other loci [33].

One mechanism by which multiple, coordinated changes in gene expression may arise is through changes in transcriptional regulators. However, changes in transcription factors can also be constrained by their pleiotropic effects on gene expression. The negative effects of pleiotropy may in some cases be eliminated by altering the regulation of a transcription factor; thereby limiting downstream changes in gene expression to specific times during development, within particular cells or tissues, or to certain environmental conditions [33], [34].

In this study, we investigated changes in gene expression and phenotype caused by a rapidly evolving transcription factor, *FZF1*. To directly target genes that have potentially accrued multiple *cis*-regulatory changes, we screened four *Saccharomyces* genomes for noncoding sequences with non-neutral patterns of divergence. *FZF1* was among the genes identified and it also shows a non-neutral pattern of amino acid divergence [35]. To examine the phenotypic consequences of *FZF1* divergence we used cross-species complementation assays and found divergence in both its coding and upstream noncoding sequence affect sulfite resistance. Whereas divergence upstream of *FZF1* affects its expression in response to sulfites, divergence in the coding region of *FZF1* affects the expression of *SSU1*, an efflux pump that mediates sulfite resistance [36]–[38]. Coincident with their effects on sulfite resistance, both the coding and noncoding regions of *FZF1* affect the expression of many other genes. Our results show how divergence in the coding and promoter region of a transcription factor affect the response to an environmental stress.

## Results

### Patterns of sequence divergence at *FZF1*

To identify promoter sequences likely to have diverged in function, we screened the noncoding sequences of four *Saccharomyces* species for accelerated substitution rates. We used a likelihood ratio test to compare a model of sequence evolution where the ratio of the noncoding to synonymous substitution rate, dNC/dS, is constant across lineages versus a model where dNC/dS is free to vary across lineages. Out of 2,539 noncoding regions tested, we identified 145 that showed significant variation in the noncoding substitution rate across species (Likelihood ratio test, P<0.05, Bonferroni corrected, Dataset S1). In these regions, a higher noncoding substitution rate in one or more lineages may be the result of loss of constraint, or in some cases, positive selection.

**Figure 1:**
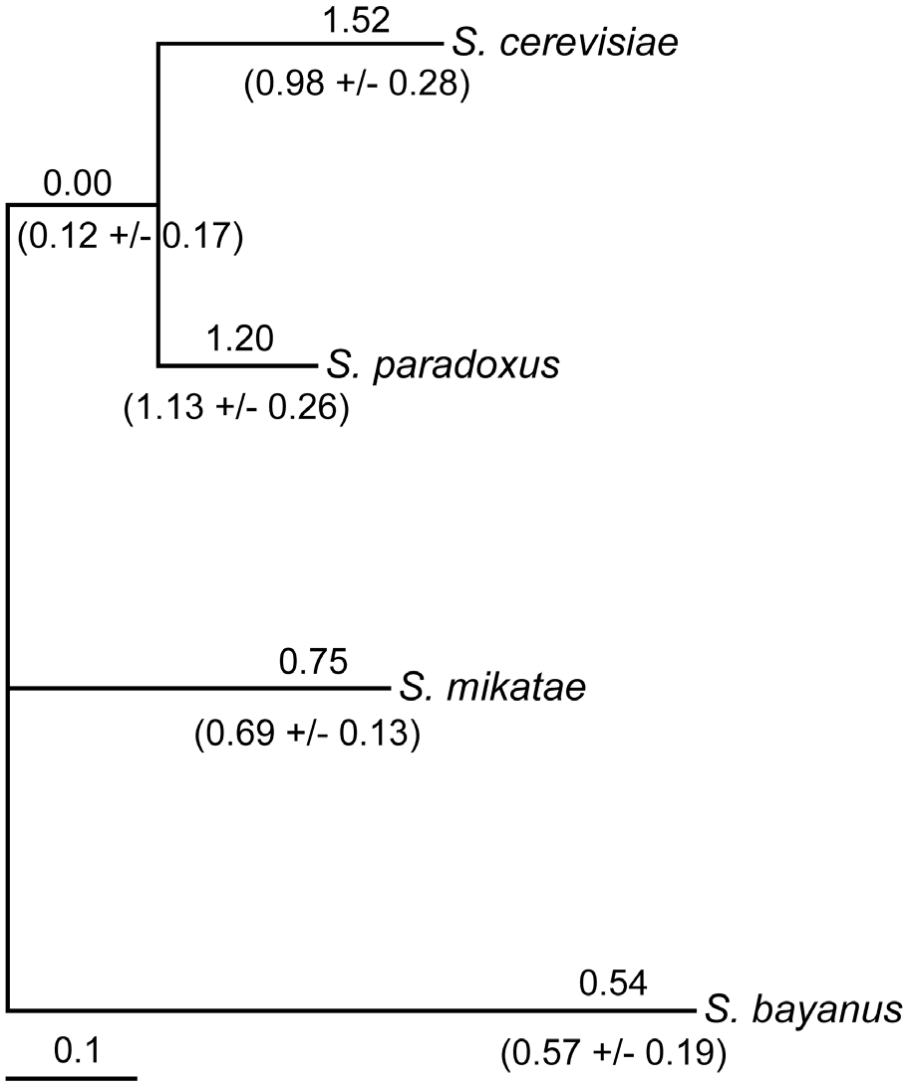
Variation in noncoding substitution rates upstream of *FZF1*. The ratio of the noncoding substitution rate upstream of *FZF1* relative to genome-wide substitution rates at fourfold degenerate sites, dNC/dS, is shown above each lineage for the original alignment. The mean and standard deviation of dNC/dS from 18 different alignments (Table S1) is shown below each lineage. dNC/dS was estimated for each lineage using an unconstrained model by maximum likelihood methods implemented in HyPhy. The tree is scaled to the fourfold synonymous substitution rate.

One of the noncoding regions that we identified lies upstream of the transcription factor *FZF1*. We selected *FZF1* for further analysis because it is known to function in sulfite resistance, a hypothesized adaptation to vineyard environments [39], and its potential role in gene expression divergence. The substitution rate upstream of *FZF1* is characterized by an accelerated rate along the lineages leading to *Saccharomyces cerevisiae* and *Saccharomyces paradoxus* relative to that along the lineages leading to *Saccharomyces mikatae* and *Saccharomyces bayanus* (Figure 1). However, previous studies have shown that signals of selection are highly dependent on the alignment [40], [41]. To determine whether the evidence for rate heterogeneity upstream of *FZF1* is dependent on the alignment used, we generated additional alignments using alternative alignment parameters and algorithms, and tested each for substitution rate heterogeneity. Both the alignment parameters and the algorithm affected the evidence for rate heterogeneity, with 9 out of 18 alignments showing evidence of rate heterogeneity (Table S1, Likelihood ratio test, P<0.05, Bonferroni corrected). Although the high substitution rate combined with uncertainty in the placement of insertions or deletions makes it difficult to know the correct alignment, dNC/dS along the *S. cerevisiae* and *S. paradoxus* lineage was consistently estimated to be greater than or equal to one (Figure 1).

The protein coding sequence of *FZF1* also shows evidence for non-neutral evolution based on a sliding window analysis of the nonsynonymous to synonymous substitution rate ratio (dN/dS) between *S. cerevisiae* and *S. paradoxus* [35]. However, caution should be taken when interpreting the results of the dN/dS test in the context of a sliding window analysis since dS can vary for a number of reasons [42]. Upon re-examination of divergence in *FZF1*, we found that the window with the signal of positive selection, dN/dS = 1.95, is characterized by a synonymous substitution rate of 0.18, which is lower than the average of 0.46 across the entire gene, and a nonsynonymous substitution rate of 0.34, which is higher than the average of 0.14 across the entire gene. Despite some uncertainty regarding the evidence for non-neutral evolution, we decided that *FZF1* was a reasonable candidate to test for functional divergence.

### The phenotypic effects of *FZF1* divergence

**Figure 2:**
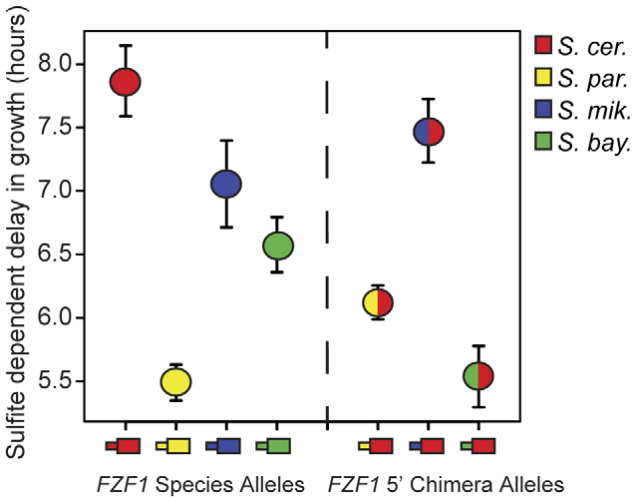
*FZF1* alleles from different species have diverged in function. The left side of the figure shows sulfite resistance of *FZF1* alleles from four different species: *S. cerevisiae* (red), *S. paradoxus* (yellow), *S. mikatae* (blue), and *S. bayanus* (green) in an *S. cerevisiae* strain background. The right side of the figure shows sulfite resistance of chimeric alleles of *FZF1* composed of the 5′ noncoding region from *S. paradoxus*, *S. mikatae* and *S. bayanus* combined with the *S. cerevisiae* coding region. Error bars show the 95% confidence interval of the mean.

*FZF1* encodes a five zinc finger transcription factor that activates the plasma membrane sulfite pump, *SSU1* [37]. Gain of function mutations in *FZF1* result in hyperactivation of *SSU1* and increased sulfite resistance [36], [38]. To determine whether *FZF1* has diverged in its ability to confer sulfite resistance, we tested *FZF1* alleles from four *Saccharomyces* species: *S. cerevisiae*, *S. paradoxus*, *S. mikitae*, and *S. bayanus*, for their ability to complement a deletion of *FZF1* in *S. cerevisiae*. The *S. cerevisiae* allele of *FZF1* showed nearly complete complementation of the *FZF1* deletion, as measured by the delay in exponential growth following sulfite treatment (Figure S1). In comparison, *FZF1* alleles from the other three species all showed a shorter delay in growth relative to that of *S. cerevisiae*, indicating that these *FZF1* alleles confer greater resistance to sulfites (Figure 2, Kruskal-Wallis test, P = 5.3×10^−13^).

To determine whether divergence in *FZF1* activity resulted from changes in its protein coding sequence or upstream noncoding sequence, we also tested chimeric constructs containing each species' *FZF1* upstream noncoding sequence combined with the *S. cerevisiae FZF1* coding sequence. These *FZF1* 5′ noncoding chimeras conferred significant differences in sulfite resistance (Figure 2, Kruskal-Wallis test, P = 2.5×10^−18^), indicating that the 5′ noncoding region alone makes a significant contribution to *FZF1* divergence. Both the *S. paradoxus - S. cerevisiae* and *S. mikatae* - S. *cerevisiae* chimeric alleles showed sulfite resistance intermediate to that of their full length parental alleles, although only the former chimera was significantly different from both parent alleles (Wilcoxon rank sum test, P = 1.9×10^−14^ for the *S. cerevisiae* parent and P = 4.2×10^−8^ for the *S. paradoxus* parent). In contrast, the *S. bayanus* 5′ noncoding region upstream of an *S. cerevisiae* coding sequence conferred greater resistance than either of the two full length parent alleles (Figure 2, Wilcoxon rank sum test, P = 4.6×10^−16^ for the *S. cerevisiae* parent and P = 2.4×10^−8^ for the *S. bayanus* parent).

### Multiple changes are responsible for divergence between *S. cerevisiae* and *S. paradoxus* alleles of *FZF1*

The *S. cerevisiae* and *S. paradoxus* alleles of *FZF1* confer the largest difference in sulfite resistance. This phenotypic divergence corresponds to the lineages showing the highest noncoding to synonymous substitution rates and the elevated nonsynonymous to synonymous substitution rate within a portion of the coding region. Thus, we further mapped the differences in sulfite resistance between the *S. cerevisiae* and *S. paradoxus FZF1* alleles.

**Figure 3:**
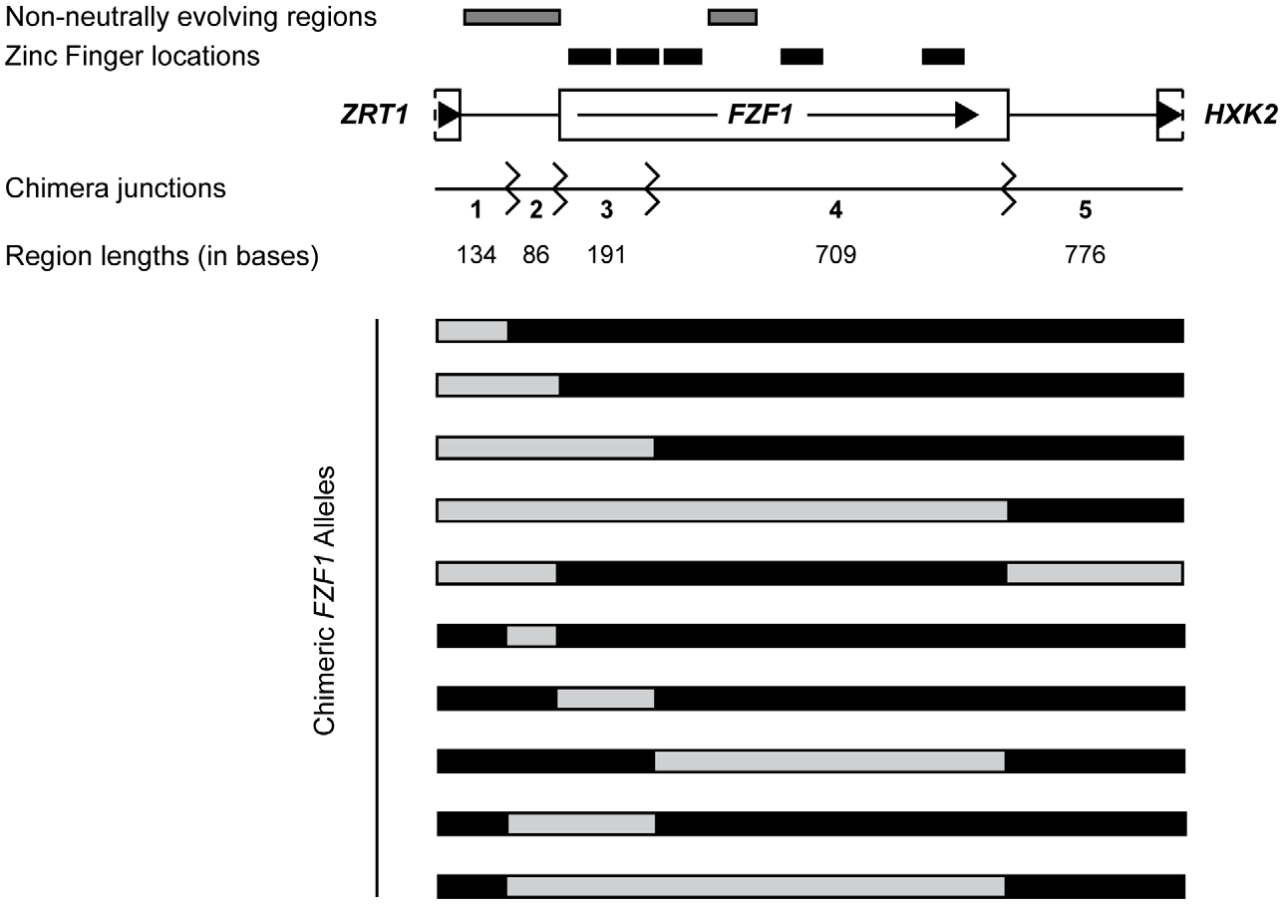
*FZF1* gene region and chimeric alleles. The *FZF1* gene region is shown along with the breakpoints used to generate reciprocal chimeric alleles. Regions with non-neutral evolution are indicated by gray boxes and the predicted zinc fingers are indicated by black boxes [73]. Region lengths between chimera junctions are given for *S. cerevisiae*. One set of chimeric alleles between *S. cerevisiae* and *S. paradoxus* are shown below. The reciprocal set is not shown.

The *S. cerevisiae FZF1* protein is 900 amino acids long and has 195 bases in the 5′ noncoding region. Between the *S. cerevisiae* and *S. paradoxus FZF1* alleles there are 67 amino acid differences and 82 differences in the 5′ noncoding region, 31 of which are insertion/deletion differences. To delineate which subset of these differences are responsible for divergence in sulfite resistance, we generated ten sets of reciprocal chimeric constructs between the two species (Figure 3). The *FZF1* chimeric breakpoints were located (1) in the middle of the 5′ noncoding region, (2) at the junction between the 5′ noncoding and the coding region, (3) in the coding region between the first zinc finger domain, known to bind DNA [37], and the region under positive selection [35], and (4) at the junction between the coding and 3′ noncoding region. Five sets of chimeric constructs contain a single region in the opposite background and the remaining sets of constructs contain five of the ten possible pairwise combinations of each region.

**Figure 4:**
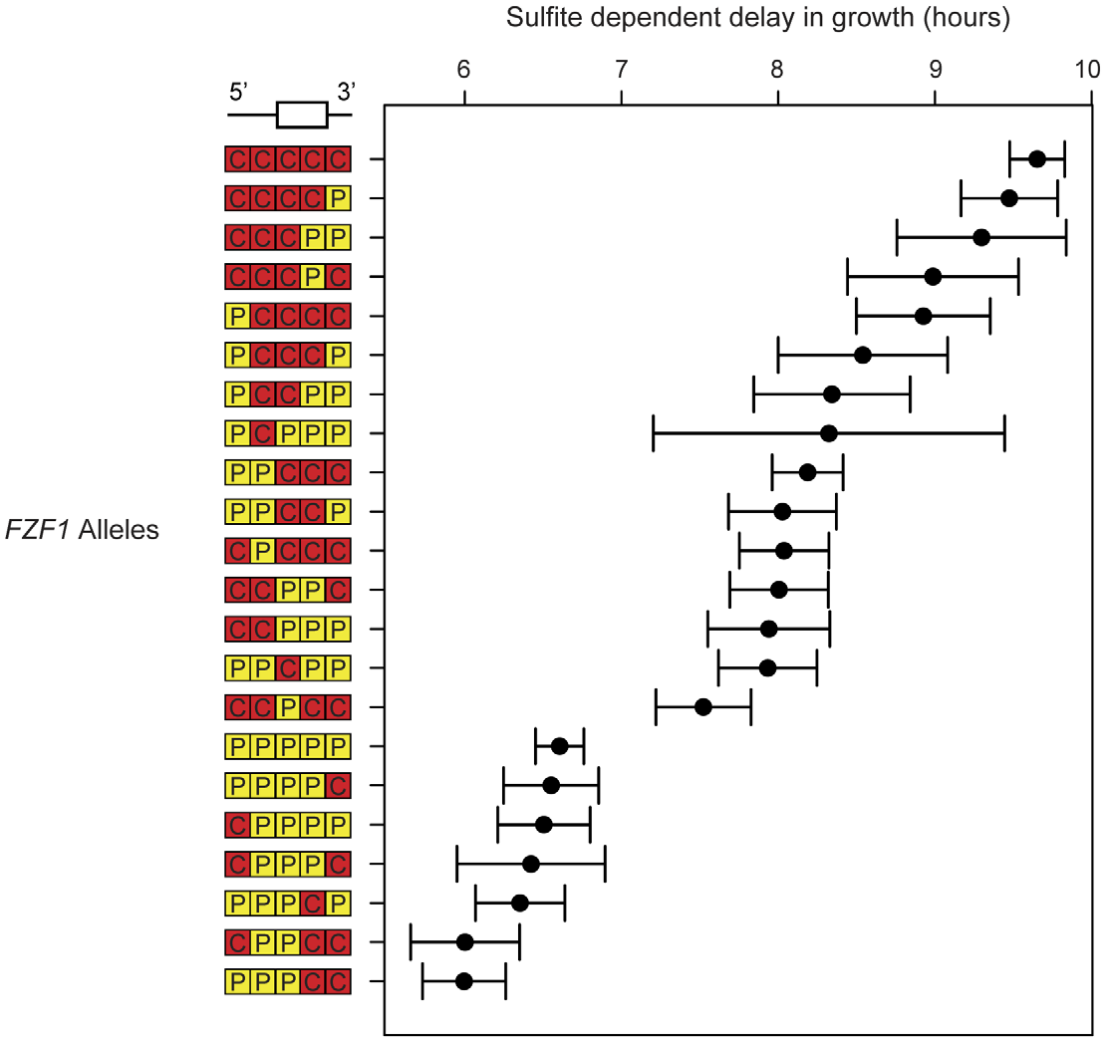
Multiple noncoding and coding changes contribute to sulfite resistance. Sulfite resistance is shown for chimeric alleles of *FZF1* from *S. cerevisiae* and *S. paradoxus*. Chimera breakpoints are shown in Figure 3 and are labeled 5′ to 3′ based on the origin of each region: *S. cerevisiae* (red, “C”) and *S. paradoxus* (yellow, “P”). Error bars show the 95% confidence interval of the mean.

Including the full length *S. cerevisiae* and *S. paradoxus* alleles of *FZF1*, the 22 constructs show a nearly continuous distribution of sulfite resistance (Figure 4). Using an additive model, the estimated effects of the first three *FZF1* regions individually account for 8.2%, 39.0%, and 49.5%, respectively, of the difference in sulfite resistance between the *S. cerevisiae* and *S. paradoxus* alleles (Table 1). The latter two regions are not statistically significant. Some of the variation in sulfite resistance can be attributed to non-additive interactions among regions. The additive model explains a total of 66% of the variance among alleles, significantly less than a model that allows for pairwise epistatic interactions, which explains 70% of the variance (Likelihood ratio test, 2Δln(L) = 56.48, 10 d.f., P = 1.7×10^−8^). However, out of all the pairwise interactions, only the interaction between the two coding regions is individually significant after correcting for multiple tests (Table 1). The interaction indicates that the two coding regions have a smaller effect in combination compared to that expected from each region individually.

**Table 1:** Estimated effects of *FZF1* divergence on sulfite resistance.

	Additive model		Epistatic model	
Region	Effect size	P-value	Effect size	P-value
1	−0.251	0.011	−0.650	3.5E−04
2	−1.189	7.2E−29	−1.283	1.7E−11
3	−1.509	2.9E−41	−1.904	2.3E−19
4	0.039	0.691	−0.423	0.020
5	0.012	0.905	−0.108	0.593
1*2			0.427	0.097
1*3			0.452	0.118
1*4			0.073	0.801
1*5			−0.107	0.676
2*3			−0.471	0.076
2*4			0.022	0.937
2*5			0.193	0.480
3*4			0.760	0.004
3*5			0.076	0.791
4*5			0.091	0.735

Effect size of each region is the estimated delay in growth due to sulfite treatment relative to the model intercept (full length *S. cerevisiae*). A star indicates an interaction between two regions. The estimated difference between the full length *S. cerevisiae* and *S. paradoxus* alleles is 3.05 hours.

### Changes in gene expression caused by *FZF1* divergence

*FZF1*-dependent changes in sulfite resistance may be mediated by changes in the expression of *FZF1* or the expression of other genes. To characterize changes in gene expression caused by *FZF1* divergence, we measured expression of *FZF1* and *SSU1*, a sulfite efflux pump activated by *FZF1* [37], [38]. Using quantitative PCR, we measured the expression of both genes before and after sulfite treatment of strains carrying an *S. cerevisiae*, *S. paradoxus*, or two reciprocal chimeric *FZF1* alleles, which divide the coding and 5′ noncoding regions of the *S. cerevisiae* and *S. paradoxus FZF1* allele.

**Figure 5:**
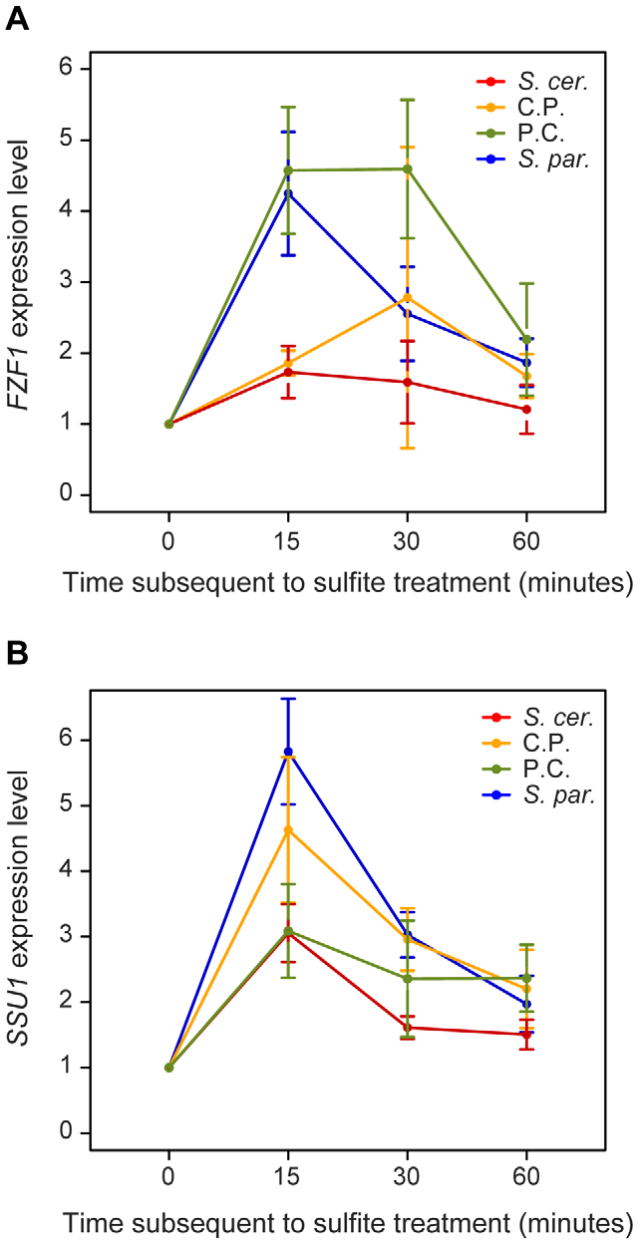
*FZF1* alleles affect the expression of both *FZF1* and *SSU1* subsequent to sulfite treatment. Expression of *FZF1* (A) and *SSU1* (B) was measured prior to and at three time-points after sulfite treatment for strains carrying four *FZF1* alleles: *S. cerevisiae* (*S. cer*, red), *S. paradoxus* (*S. par*, blue), *S. cerevisiae* 5′ noncoding with *S. paradoxus* coding (C.P., gold), *S. paradoxus* 5′ noncoding and *S. cerevisiae* coding (P.C., green). Expression levels were normalized to the 0 time-point. Each point is the mean of 3–4 individual observations. Error bars represent the 95% confidence interval of the mean.

All of the *FZF1* alleles increased in expression following sulfite treatment. However at time-points 15, 30 and 60 minutes after sulfite treatment, the *FZF1* alleles with an *S. paradoxus* promoter were expressed at higher levels than those containing an *S. cerevisiae* promoter (Wilcoxon rank sum test, P = 6.7×10^−9^, P = 1.7×10^−4^, P = 0.008, respectively, Figure 5A). No significant differences were found due to the *FZF1* coding region alone from the two species. Yet, 30 minutes after sulfite treatment, the two *FZF1* alleles with the *S. paradoxus* promoter showed significant differences in expression; the allele with an *S. cerevisiae* coding region remained at a higher level relative to the allele with an *S. paradoxus* coding region (Wilcoxon rank sum test, P = 0.0012). Similarly, the *FZF1* allele with an *S. cerevisiae* promoter and *S. paradoxus* coding region showed higher expression at the 30 minute time-point relative to the full length *S. cerevisiae* allele, although this difference was not significant (Wilcoxon rank sum test, P = 0.15). Differences in gene expression that depend on changes within a coding region have previously been found in yeast [43] and could result from feedback regulation.

The *FZF1* alleles also caused an increase in *SSU1* expression after sulfite treatment (Figure 5B). Unlike *FZF1* expression, *SSU1* expression primarily depended on the origin of the *FZF1* coding region. For both the 15 and 30 minute time-points, *FZF1* alleles containing the *S. paradoxus* coding region caused higher levels of *SSU1* expression relative to those containing the *S. cerevisiae* coding region (Wilcoxon rank sum test, P = 1.15×10^−5^, P = 8.94×10^−6^, respectively). No significant differences in *SSU1* expression were found as a result of the *FZF1* 5′ noncoding region alone.

If *FZF1*-dependent differences in sulfite resistance are mediated by activation of *FZF1* and *SSU1*, they may also be influenced by levels of *FZF1* and *SSU1* expression prior to sulfite treatment. Immediately prior to sulfite treatment, *FZF1* alleles with the *S. cerevisiae* coding sequences were expressed at 1.5-fold higher levels than those with the *S. paradoxus* coding sequence (Wilcoxon rank sum test, P = 1.3×10^−6^). The 5′ noncoding region caused no significant differences in *FZF1* expression prior to sulfite treatment. In comparison, expression of *SSU1* prior to sulfite treatment was 1.09-fold higher for *FZF1* alleles containing the *S. cerevisiae* coding region and 1.12-fold higher for *FZF1* alleles containing the *S. cerevisiae* 5′ noncoding region relative to the corresponding *S. paradoxus* regions (Wilcoxon rank sum test, P = 0.011, P = 6.5×10^−4^, respectively). Because the *S. paradoxus* allele of *FZF1* causes higher levels of sulfite resistance, levels of *FZF1* expression prior to sulfite treatment do not appear to be related to sulfite resistance.

The effect of *FZF1* divergence on *SSU1* expression suggests that *FZF1* may also affect the expression of other genes. To examine this possibility, we measured genome-wide changes in expression caused by the *S. cerevisiae* and *S. paradoxus FZF1* alleles and the two reciprocal 5′ noncoding chimeras. Gene expression was measured using microarrays before and 15 minutes after addition of sulfites. Out of 6127 open reading frames queried, 655 showed *FZF1*-dependent differences in expression across both time-points and 648 showed *FZF1*-dependent differences in expression that varied by time-point (ANOVA, P<0.01 for both). For both tests, permutation resampling of the data indicated a false discovery rate of 9.8%. Out of the combined set of 1,096 genes that showed *FZF1*-dependent differences in expression, 87% showed significant changes following sulfite treatment (ANOVA, P<0.01), of which 219 and 271 showed a >2-fold decrease and increase, respectively, in expression following sulfite treatment. Consistent with other studies of the stress response [44], [45], many of the genes that decreased in expression are involved in ribosome biogenesis (64 genes) and many of the genes that increased in expression are involved in oxidation reduction (51 genes) and response to abiotic stimulus (49 genes)(Dataset S2). Overall, strains carrying the *S. cerevisiae FZF1* allele showed more pronounced changes in expression than those carrying the *S. paradoxus* allele (Figure S2), consistent with the possibility that many of the expression differences are not due to direct differential activation or repression by *FZF1*, but rather a consequence of downstream differences in sulfite resistance initiated by *FZF1*. A small number of genes, including *SSU1*, showed a larger increase in expression in strains carrying the *S. paradoxus* compared to the *S. cerevisiae FZF1* allele. Excluding two putative genes, *SSU1* showed the largest differences in expression between the *S. cerevisiae* and *S. paradoxus* alleles at 15 minutes and was one of the most significant *FZF1*-dependent differences across both time-points.

**Figure 6:**
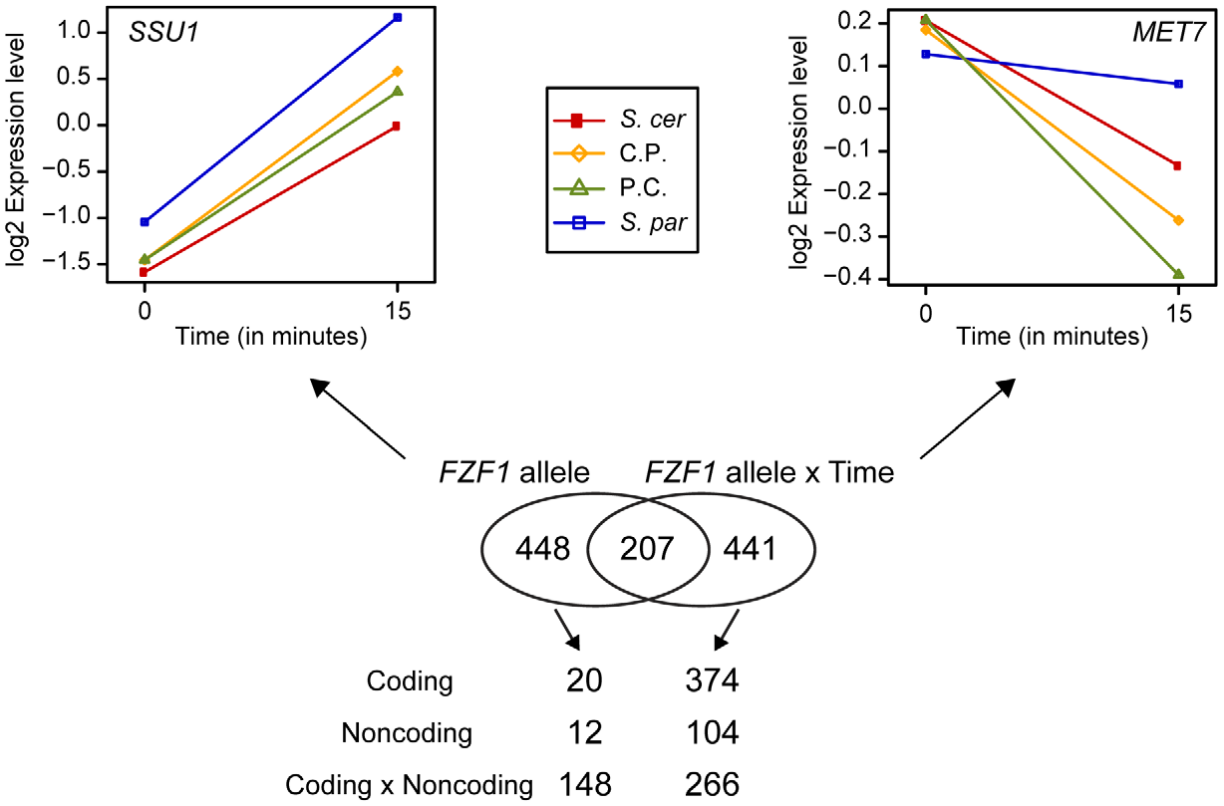
Changes in gene expression caused by *FZF1* divergence. A Venn diagram of the number of genes with expression differences that depended on the *FZF1* allele or an interaction between the *FZF1* allele and time. An example of a gene with expression differences due to the *FZF1* allele alone (*SSU1*) or an interaction between the *FZF1* allele and time (*MET7*) are shown above the Venn diagram: *S. cerevisiae* (*S. cer*, red), *S. paradoxus* (*S. par*, blue), *S. cerevisiae* 5′ noncoding with *S. paradoxus* coding (C.P., gold), *S. paradoxus* 5′ noncoding and *S. cerevisiae* coding (P.C., green). The number of genes with expression differences that can be attributed to the coding region, upstream noncoding region or an interaction between the two regions is shown below the Venn diagram.

*FZF1*-dependent changes in gene expression may be caused by protein coding changes or by regulatory changes in the *FZF1* 5′ noncoding region. To distinguish between these possibilities, we classified *FZF1*-dependent expression changes into those that can be attributed to the 5′ noncoding region, coding region, or an interaction between the two regions. Most of the genes that showed *FZF1*-dependent differences in gene expression across both time-points were characterized by an interaction between the coding and 5′ noncoding regions (ANOVA, P<0.01, Figure 6). Interestingly, in many cases, the chimeric alleles caused these genes to be expressed at higher or lower levels compared to both of the full length alleles of each species. In contrast, most of the genes showing allele-specific differences in gene expression that varied by time-point were characterized by effects that depended on the coding region of *FZF1* (ANOVA, P<0.01, Figure 6). Together, these results suggest that both the *FZF1* coding and 5′ noncoding region contribute to downstream changes in gene expression.

## Discussion

Identification of genes that have diverged in function between species is a key element to understanding species' diversity and evolution. Divergence in transcription factors are of particular interest as they can coordinately regulate the expression of many changes, but by doing so may be limited in how they can evolve. In this study, we used patterns of non-neutral sequence evolution to identify genes likely to have diverged in their regulation. We investigated one candidate, *FZF1*, by testing species-specific alleles for their ability to complement a deletion of *FZF1* in *S. cerevisiae*. We found that *FZF1* has diverged in its ability to confer resistance to sulfites, and used chimeric constructs to show that divergence in sulfite resistance is due to changes in multiple coding and upstream noncoding regions. Finally, we found that divergence at *FZF1* affects the expression of *FZF1*, *SSU1* and many other genes. Our results provide insight into how both phenotypic and regulatory divergence is caused by evolution of a transcription factor.

### Identification of *FZF1* and evidence for non-neutral evolution

We identified *FZF1* based on a genome-wide screen for patterns of non-neutral divergence. *FZF1* shows evidence of non-neutral divergence in its promoter region based on an accelerated substitution rate in some lineages but not others. In the coding region, evidence of non-neutral divergence is also present and is based on an elevated ratio of nonsynonymous to synonymous substitutions. However, upon closer examination we found a number of uncertainties regarding the evidence for non-neutral patterns of divergence. In the noncoding region, the evidence for substitution rate heterogeneity depends on the alignment. In the coding region, the cause of the elevated nonsynonymous to synonymous substitution rate is ambiguous because the synonymous substitution rate decreases in the same region that the nonsynonymous substitution rate increases. Interestingly, the strongest evidence for non-neutral evolution comes from divergence between the *S. cerevisiae* and *S. paradoxus* alleles, which also show the greatest difference in sulfite resistance. Thus, the pattern of divergence for *FZF1* is at least consistent with non-neutral evolution. With respect to a potential cause of non-neutral divergence, both positive selection and loss of constraint can result in elevated substitution rates. However, loss of constraint by itself does not provide a good explanation for the loss of sulfite resistance along the *S. cerevisiae* lineage and the gain of sulfite resistance along the *S. paradoxus* lineage relative to the intermediate levels of sulfite resistance in *S. mikatae* and *S. bayanus*.

While patterns of non-neutral divergence led us to test *FZF1* alleles for functional divergence, the value of such an approach remains difficult to assess. First, the evidence for non-neutral evolution is not definitive. Second, we only tested a single candidate. Third, the coding region with the largest effect on sulfite resistance does not include the region with evidence for non-neutral evolution. One factor that may be critical in selecting candidates is whether there is a clearly defined phenotype to test. Many of the other genes that exhibit substitution rate heterogeneity are known to impact a variety of phenotypes, making it difficult to know which one to test. Testing *FZF1* was facilitated by its narrowly defined function in sulfite resistance. Thus, while some fascinating examples have emerged, e.g. [46], further work is needed to evaluate whether non-neutral patterns of divergence provide an effective screen for genes that have diverged in function.

### Evolution of *FZF1* through multiple coding and noncoding changes

Chimeric *FZF1* alleles from *S. cerevisiae* and *S. paradoxus* indicate that both upstream noncoding regions and the first coding region make additive contributions to divergence in *FZF1* activity. A second coding region, including the region with the elevated nonsynonymous to synonymous substitution rate, contributes an epistatic effect through interaction with the other coding region. The number of regions underlying sulfite resistance is likely dependent on how we identified *FZF1*. Tests of neutrality based on rate heterogeneity and dN/dS only indicate deviations from expected rates of divergence based on multiple substitutions. The accumulation of multiple changes at a single locus has also been found in other studies of interspecific differences [22]–[26], so it may not be an uncommon result when multiple regions are individually tested.

A limitation of our study is that we only quantified the effects of five regions and did not narrow their effects to individual substitutions. This limitation is in part due to the sensitivity of our sulfite resistance assay. As such, we did not determine whether the regions with the largest effect are caused by single or multiple substitutions, and whether there are epistatic effects between substitutions within a region. Further dissection of *FZF1* divergence is needed to more accurately quantify the number, effect size and interactions among mutations affecting sulfite resistance.

### Evolution of gene regulation

Transcription factors are often posited to be highly constrained during evolution due to their pleiotropic effects on the expression of other genes [47]. As such, many efforts to understand the evolution of gene regulation have focused on the evolution of *cis*-regulatory sequences rather than on *trans*-acting factors, e.g. [12], [48]. While changes in the expression of transcriptional regulators is hypothesized to be an important mode of evolutionary change [34], protein coding changes may also be important, e.g. [49]. We find that divergence in both the regulatory and coding sequence of *FZF1* affects sulfite resistance and causes numerous downstream changes in gene expression. This raises the question of whether there have been any constraints on *FZF1* divergence due to pleiotropy.

If *FZF1* has been constrained by pleiotropy there must, at least under certain circumstances, be negative consequences to changes in *FZF1* activity. Increased levels of *FZF1* activity could reduce fitness in the absence of sulfites or after other exposures that activate *FZF1*, such as nitric oxide treatment [50]. The observation that the more potent *S. paradoxus FZF1* allele is expressed at lower levels in the absence of sulfites provides some support for the idea that high levels of *FZF1* activity may not always be advantageous. Assuming that there is some cost to constitutive increases in *FZF1* activity, there are a number of ways in which this cost could be small enough to overcome or even eliminated.

One consideration is that *SSU1* expression is likely the major determinant of sulfite resistance and so the benefit of increased *SSU1* expression may outweigh any costs. In support of this possibility, *SSU1* overexpression is able to rescue the effect of an *FZF1* deletion (Figure S3) [38]. However, the expression of other genes may also be involved in sulfite resistance since divergence upstream of *FZF1* affects sulfite resistance but only has a small, insignificant effect on *SSU1* expression. Thus, coding changes in *FZF1* that increase *SSU1* expression may have outweighed any costs under other conditions, or may have been facilitated by lower levels of *FZF1* expression in the absence of sulfites.

Another explanation for the lack of constraints on *FZF1* divergence is compensatory changes in genes regulated by *FZF1*. In this scenario, slight changes in *FZF1* activity may be compensated by *cis*-regulatory mutations in those *FZF1* regulated genes where changes in gene expression are deleterious. A number of empirical studies have shown that transcription factors bind different targets between closely related species and even within species due in part to *cis*-regulatory sequence changes [11], [51]–[53]. Thus, it is also possible that *cis*-regulatory sequence evolution may have accommodated divergence in *FZF1* activity.

A third explanation, suggested by the finding that transcription factors with few targets are less likely to be constrained by pleiotropy [54], is that *FZF1* has few transcriptional targets and so is not greatly constrained by pleiotropy. In response to exogenously supplied nitric oxide, activation of only a small set of five genes, including *SSU1*, was found to specifically depend on the presence of *FZF1* [50]. Another study found 21 upregulated and 37 downregulated genes two hours after sulfite treatment [55]. We found 1,096 *FZF1*-dependent expression changes, most of which showed the same direction of response to sulfite and only differed in magnitude. The observation that the sulfite-sensitive *S. cerevisiae FZF1* allele caused more pronounced changes in gene expression relative to the *S. paradoxus* allele (Figure S2) is consistent with *FZF1* causing indirect changes in gene expression mediated by its effects on sulfite resistance rather than by direct activation or repression of these genes. Furthermore, we found no enrichment of the *FZF1* motif identified in the *SSU1* promoter (TATCGTAT and CAACAA, [37]), defined by protein microarrays (CTGCTA, [56]), or by promoter bashing and response to nitrosative stress (YGSMNMCTATCAYTTYY, [50]) within the 271 genes showing a 2-fold significant increase in expression following sulfite treatment. Thus, most of the changes in gene expression that we observed may be an indirect consequence of a sulfite-induced stress response rather than a consequence of changes in direct targets of *FZF1*.

Regardless of the mechanism, the concentration of multiple sequence changes in *FZF1* suggests that it may have evolved without many genetic, functional or evolutionary constraints. However, the apparent absence of constraints could be a consequence of low basal levels of *FZF1* expression. Under this scenario, changes in *FZF1* regulation may have facilitated changes within its protein coding sequence.

### Evolution of sulfite resistance

Even though *FZF1* has diverged in its ability to confer resistance to sulfites, its impact on the evolution of sulfite resistance is hard to know. While there is substantial variation in sulfite resistance within and between species (Figure S4), divergence at other loci may be responsible for most differences in sulfite resistance and could compensate for any changes in *FZF1*. Within *S. cerevisiae*, variation in sulfite resistance is associated with a reciprocal translocation upstream of *SSU1* that is more frequent in vineyard and wine strains than strains derived from other sources [39], [57]. The inferred loss of sulfite resistance conferred by changes in *FZF1* along the lineage leading to *S. cerevisiae*, combined with the gain of sulfite resistance due to the translocation within some strains of *S. cerevisiae*, suggests that the evolution of sulfite resistance among species is not simple and compensatory changes may be involved.

### Conclusions

In this study we find substantial divergence in function within the coding and upstream noncoding region of *FZF1*. Our finding that multiple regions underlie divergence in sulfite resistance is not unexpected given the patterns of non-neutral evolution, but differs from other studies that identify single changes of large effect based on genetic mapping or candidate gene approaches [14]. The contribution of both noncoding and coding regions to differences in sulfite resistance suggests that the distinction between evolution in noncoding and coding regions may be less important than the degree to which a gene has the capacity to evolve, unencumbered by constraints on its other functions [33]. In conclusion, our work supports a model whereby both gene expression and phenotypic divergence can be attributed to multiple mutations throughout the regulatory and protein-coding region of a single gene.

## Materials and Methods

### Screen for noncoding regions with substitution rate heterogeneity

*S. cerevisiae*, *S. paradoxus*, *S. mikatae*, and *S. bayanus* noncoding regions [58] were tested for substitution rate heterogeneity using a likelihood ratio test implemented using HyPhy [59]. The likelihood ratio test was used to compare a constrained model with a single substitution rate across lineages to an unconstrained model where each lineage was allowed to have a different substitution rate. For both models we used the HKY85 substitution model implemented in HyPhy, the known phylogenetic relationship among the species, and either a single parameter (constrained) or branch-specific parameters (unconstrained) for the ratio of the noncoding substitution rate at the locus of interest to the substitution rate at four-fold degenerate sites across the genome. Noncoding alignments were removed if the total length of insertion/deletions was more than 15% of the length of the entire alignment. While this filter eliminated the noncoding region upstream of *FZF1*, we had already initiated our functional analysis of *FZF1* based on preliminary rate heterogeneity results and so retained it in our list of candidates. To examine whether substitution rate heterogeneity upstream of *FZF1* depends on the alignment, we aligned the 5′ noncoding region using 6 alignment programs: Clustalw [60], MUSCLE [61], TCOFFEE [62], MAFFT [63], PRANK [64], and DCA [65]. The resulting alignments were tested for rate heterogeneity using the likelihood ratio test described above. For the coding sequence of *FZF1*, a sliding window analysis of dN/dS was performed for *FZF1* using the K-estimator software [66] as described in Sawyer and Malik (2006). K-estimator uses Monte Carlo simulations to estimate the confidence intervals for estimates of dN/dS.

### Strain construction

*FZF1* was deleted in YJF173 (S288c-background, *Mat a*, *ho*, *ura3-52*) using the KANMX deletion cassette [67]. *FZF1* alleles were integrated into this strain at the *ura3* locus by amplifying the entire *FZF1* gene region, including the entire 5′ and 3′ noncoding regions along with 25 bases of *ZRT1* and 45 bases of *HXK2*, using primers with homology to pRS306 and transforming the product along with the yeast integrative plasmid, pRS306 [68]. Integration of these constructs at the *ura3* locus was achieved by selection on plates lacking uracil and each transformant was confirmed by PCR. Chimeras were generated using the same procedure but with *FZF1* regions amplified from different species. The aligned ATG start site was used for all chimeras divided between the 5′ noncoding region and the coding region. A mutation of an alternate *FZF1* start site in the *S. paradoxus FZF1* allele did not significantly alter sulfite resistance compared to the non-mutated counterpart (data not shown). A subset of 2–5 transformants were sequenced to ensure that at least one transformant per construct contained no mutations.

### Media and growth conditions

All experiments were conducted using YPD+TA (1% yeast extract, 2% peptone, 2% dextrose, 75 mM L-tartaric acid buffered to pH 3.5) [69]. Sulfite resistance was measured by comparing growth in the presence and absence of sodium sulfite. Strains were grown overnight in YPD+TA, diluted 1∶1000 in YPD+TA, grown for 3 hours, treated with either water or sodium sulfite (final concentration 0.7–0.9 mM sodium sulfite), and then grown for 20 hours in an iEMS plate reader at 30° with 1200 rpm shaking (model no. 1400; Thermo Lab Systems, Helsinki, Finland). For each strain, the sulfite-dependent delay in growth was determined by comparing the time at which maximum growth rate was observed for strains treated with sulfite relative to a water-treated control [70]. For each *FZF1* construct, 4 to 8 independent transformants were phenotyped. To compare the sulfite-dependent delay in growth within and between yeast species, three replicate measurements were obtained for 6 *S. cerevisiae* strains: S288c (source: laboratory, obtained from: D. Botstein), YPS163 (source: oak exudate, United States, obtained from: P. Sniegowski), M8 and M33 (source: vineyard, Italy, obtained from R. Mortimer), YJM440 (source: clinical, United States, obtained from: J. McCusker), K9 (source: saké, Japan, obtained from: Nami Goto-Yamamoto), and five *S. paradoxus* strains: YPS138 (source: oak soil, United States), N17 (source: oak exudate, Russia), N44 (source: oak exudate, Russia), Y7 (source: oak bark, United Kingdom), and NRRL Y-17217 all obtained from G. Litti and E. Louis. Additional yeast species included: *S. mikatae* (IFO1815, obtained from: E. Louis), *S. bayanus* (NRRL Y-11845, obtained from: C. Kurtzman, ARS Culture Collection), *Saccharomyces castellii* (NRRL Y-12630, obtained from: M. Johnston), *Saccharomyces kluyverii* (NRRL Y-12651, obtained from: M. Johnston), and *Kluyveromyces lactis* (FM423, a haploid MAT á strain obtained from M. Johnston). All strains are diploid except as noted.

### Analysis of sulfite resistance

Differences in sulfite resistance between species and species' chimeras were normalized for day effects and tested for significance using the nonparametric Kruskal-Wallis test. Pairwise differences between constructs were examined using the nonparametric Wilcoxon rank sum test with Bonferroni correction.

Differences in sulfite resistance among *S. cerevisiae* - *S. paradoxus* chimeric constructs of *FZF1* were measured using linear mixed effect (lme) models to account for repeated measurements of the same construct. Sulfite resistance of each construct was measured three times and measurements on different days were standardized by a Z-score transformation. Sulfite resistance was fit to two models. The first model assumes each region from *S. cerevisiae* or *S. paradoxus* makes an additive contribution to differences in sulfite resistance: *sulfite resistance = region1+region2+region3+region4+region5+(error | batch)+error*, where each region has an effect that depends on the species the region came from and (error | batch) models random effects due to measurement of the same construct in different batches (96-well plates). The second model builds on the first model but adds in all pairwise interactions between regions: *sulfite resistance = (region1+region2+region3+region4+region5)∧2+(error | batch)+error*. The fit of the two models was compared using a likelihood ratio test with 10 degrees of freedom since the first and second models have 8 and 18 degrees of freedom, respectively. The percent variance explained by each model was calculated by R^2^ = 1−exp(−LR/n), where n is the sample size and LR is the likelihood ratio statistic defined by twice the difference in the log likelihood of the alternative relative to the null model [71]. The null model was fit using only an intercept: *sulfite resistance = (error | batch)+error*. For lme P-values, we tested whether the assumptions of the test were violated and resulted in inaccurate P-values by repeatedly permuting the data labels to obtain the distribution of P-values expected by chance. The permuted data showed no evidence for inaccurate P-values.

### Gene expression analysis

Gene expression was measured using four independent transformants of each *FZF1* construct. Strains were resuspended in YPD+TA at an OD600 of 0.25 from an overnight YPD+TA culture and grown in 100 mL cultures at 30°C, 200 rpm. After 3 hours, each culture was sampled at 0, 15, 30 and 60 minutes after addition of sodium sulfite to a final concentration of 1 mM. Cells were centrifuged, washed and frozen in a dry ice/ethanol bath and stored at −80°C. RNA was isolated and cDNA prepared using Qiagen's RNaeasy Mini Kit and Omniscript RT Kit, respectively (Valencia, CA).

Quantitative PCR was used to measure expression of *FZF1* and *SSU1*. A 20-fold dilution of cDNA reactions was used for the real-time PCR assays with gene specific primers and Strategene's Brilliant II SYBR Green QPCR Master Mix (Santa Clara, CA). Expression was assayed on Stratagene's MX3000P QPCR machine. For *FZF1*, species-specific primers were used and a plate specific correction factor, estimated for each plate from quantitative PCR measurements of DNA extracted from a heterozygous strain containing both the *S. cerevisiae* and *S. paradoxus FZF1* alleles, was used to account for the difference in PCR efficiency between the *S. cerevisiae* and *S. paradoxus* primers. Data were mean normalized for day and batch effects and expression levels were measured relative to *ACT1*. The Wilcoxon rank sum test with Bonferroni correction was used to identify significant differences in expression due to *FZF1* alleles.

Genome-wide measurements of gene expression were obtained using Agilent Technologies (Santa Clara, CA) yeast (V2) gene expression microarrays (8×15K, Catalog number: G4813A-016322) following the manufacturers protocols. Sample labeling, hybridization and microarray scanning was conducted by the Expression and Genotype Core at Washington University's Genome Center. Gene expression was measured for three independent replicates at the 0 and 15 minute time-points. Each sample was compared to a reference made up of a pool of all RNA samples. Expression data was deposited in the GEO database under accession GSE35308. After median normalization of each microarray, differences in gene expression were tested using an analysis of variance (ANOVA) with the model: expression = allele*time+technical replicate+error, where *allele* measures the effect of the different *FZF1* alleles, *time* measures the effect of each time-point, and *technical replicate* accounts for differences between replicated features on the microarray. The rate of false positives was estimated by permuting the sample labels 100 times and repeating the analysis. For each gene showing a significant difference in expression, a second ANOVA was performed to identify expression changes that could be attributed to the coding or 5′ noncoding region or an interaction between the two regions. For genes showing expression differences that depended on the *FZF1* construct we used the model: expression = noncoding*coding+error, and for genes showing differences that depended on an interaction between the *FZF1* construct and time we used the model: expression = noncoding*coding*time+error. Gene sets enriched for gene ontology (GO) categories were identified using DAVID [72].

## Supporting Information

Dataset S1 Noncoding regions showing evidence for substitution rate heterogeneity across four *Saccharomyces* species.
(XLS)

Dataset S2 Differentially expressed genes and associated GO terms.
(XLS)

Figure S1 Deletion of *FZF1* causes a sulfite dependent delay in growth that is rescued by integration of *FZF1* at the *URA3* locus. A. Sulfite resistance was measured by a sulfite-dependent delay in growth based on the time at which maximum growth rate was achieved in the presence (solid line) or absence (dashed line) of sulfite. Growth curves are shown for an *FZF1* deletion strain (red) and the same strain with an *S. cerevisiae* allele of *FZF1* integrated at the *URA3* locus (black). Lines represent the mean of three replicates. B. Sulfite resistance of a wildtype *S. cerevisiae* strain (WT), an *FZF1* deletion strain, and a strain carrying the *S. cerevisiae FZF1* allele at the *URA3* locus (*S. cer* rescue). The integration of an *S. cerevisiae FZF1* allele reduced the sulfite dependent delay of growth caused by deletion of the endogenous *FZF1* allele to similar levels as the parental *S. cerevisiae* strain. Bars represent the 95% confidence interval of the mean delay in growth.
(PDF)

Figure S2 Sulfite-dependent changes in gene expression are larger for the *S. cerevisiae FZF1* allele relative to the *S. paradoxus* allele. The log2 fold-change in expression as a result of sulfite treatment is shown for the *S. cerevisiae* (*S. cer*), *S. paradoxus* (*S. par*) and two 5′ noncoding chimeric alleles of *FZF1* (C.P., P.C.). Box plots are shown for the 149 up-regulated genes (>4-fold, P<0.01) in panel A and 83 down-regulated genes (>4-fold, P<0.01) in panel B.
(PDF)

Figure S3 *SSU1* overexpression increases sulfite resistance in wildtype and *FZF1* deletion strains. A–D. Sulfite resistance in *S. cerevisiae* wildtype (A) and an *FZF1* deletion strain (B) to a range of sulfite concentrations. *SSU1* overexpression significantly increases resistance to higher sulfite concentrations in wildtype *S. cerevisiae* (C) and *FZF1* deletion strains (D). Lines represent the mean of three replicates. E. Strains overexpressing *SSU1*, show a reduced sulfite dependent delay in growth at 0.3 mM sodium sulfite compared to the parental strains. Bars represent the 95% confidence interval of the mean.
(PDF)

Figure S4 Variation in sulfite resistance within and between yeast species. Sulfite resistance is shown for 6 strains of *S. cerevisiae*, 5 strains of *S. paradoxus*, and a single strain of *S. mikatae*, *S. castellii* and *K. lactis*. Error bars show the 95% confidence interval of the mean. The error bars are within the circles for M8, M33 and YPS138. Sulfite resistance was also measured for a strain of *S. kluyverii* and *S. bayanus*, but the sulfite-dependent delay in growth could not be calculated since the strains grew in the presence of water (control) but not in the presence of sulfite. The wine strain M8 has the known translocation upstream of *SSU1* that increases sulfite resistance [39], [57], [74].
(PDF)

Table S1 Evidence for rate heterogeneity upstream of *FZF1* depends on the alignment.
(XLS)
