# Supplementary material for: Divergence of the Yeast Transcription Factor FZF1 Affects Sulfite Resistance
Source: PLoS Genet. 2012 Jun 14;8(6):e1002763. doi: 10.1371/journal.pgen.1002763 (PMC3375221; doi:10.1371/journal.pgen.1002763)
Supplement: Figure S1 — Deletion of FZF1 causes a sulfite dependent delay in growth that is rescued by integration of FZF1 at the URA3 locus. A. Sulfite resistance was measured by a sulfite-dependent delay in growth based on the time at which maximum growth rate was achieved in the presence (solid line) or absence (dashed line) of sulfite. Growth curves are shown for an FZF1 deletion strain (red) and the same strain with an S. cerevisiae allele of FZF1 integrated at the URA3 locus (black). Lines represent the mean of three replicates. B. Sulfite resistance of a wildtype S. cerevisiae strain (WT), an FZF1 deletion strain, and a strain carrying the S. cerevisiae FZF1 allele at the URA3 locus (S. cer rescue). The integration of an S. cerevisiae FZF1 allele reduced the sulfite dependent delay of growth caused by deletion of the endogenous FZF1 allele to similar levels as the parental S. cerevisiae strain. Bars represent the 95% confidence interval of the mean delay in growth. (PDF) [file pgen.1002763.s003.pdf]

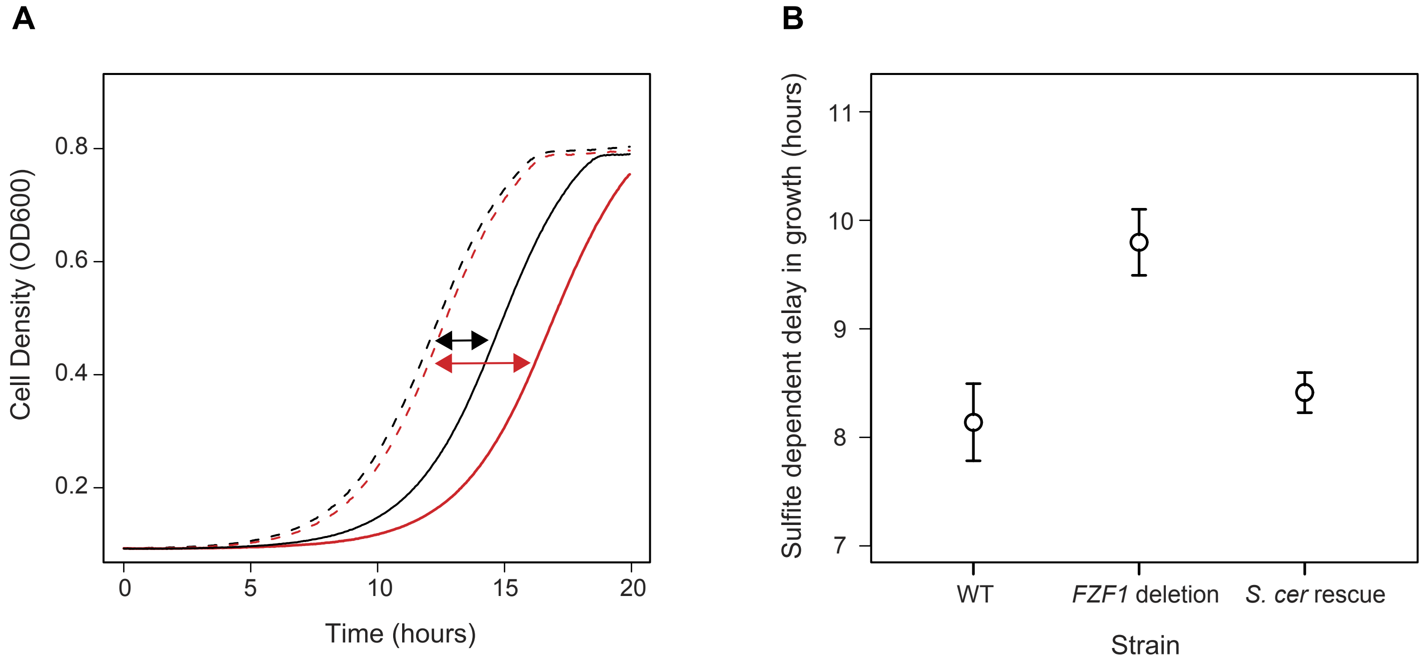

Figure S1: Deletion of *FZF1* causes a sulfite dependent delay in growth that is rescued by integration of *FZF1* at the *URA3* locus.

A. Sulfite resistance was measured by a sulfite-dependent delay in growth based on the time at which maximum growth rate was achieved in the presence (solid line) or absence (dashed line) of sulfite. Growth curves are shown for an *FZF1* deletion strain (red) and the same strain with an *S. cerevisiae* allele of *FZF1* integrated at the *URA3* locus (black). Lines represent the mean of three replicates. B. Sulfite resistance of a wildtype *S. cerevisiae* strain (WT), an *FZF1* deletion strain, and a strain carrying the *S. cerevisiae FZF1* allele at the *URA3* locus (*S. cer* rescue). The integration of an *S. cerevisiae FZF1* allele reduced the sulfite dependent delay of growth caused by deletion of the endogenous *FZF1* allele to similar levels as the parental *S. cerevisiae* strain. Bars represent the 95% confidence interval of the mean delay in growth.
