# Supplementary material for: Divergence of the Yeast Transcription Factor FZF1 Affects Sulfite Resistance
Source: PLoS Genet. 2012 Jun 14;8(6):e1002763. doi: 10.1371/journal.pgen.1002763 (PMC3375221; doi:10.1371/journal.pgen.1002763)
Supplement: Figure S2 — Sulfite-dependent changes in gene expression are larger for the S. cerevisiae FZF1 allele relative to the S. paradoxus allele. The log2 fold-change in expression as a result of sulfite treatment is shown for the S. cerevisiae (S. cer), S. paradoxus (S. par) and two 5′ noncoding chimeric alleles of FZF1 (C.P., P.C.). Box plots are shown for the 149 up-regulated genes (>4-fold, P<0.01) in panel A and 83 down-regulated genes (>4-fold, P<0.01) in panel B. (PDF) [file pgen.1002763.s004.pdf]

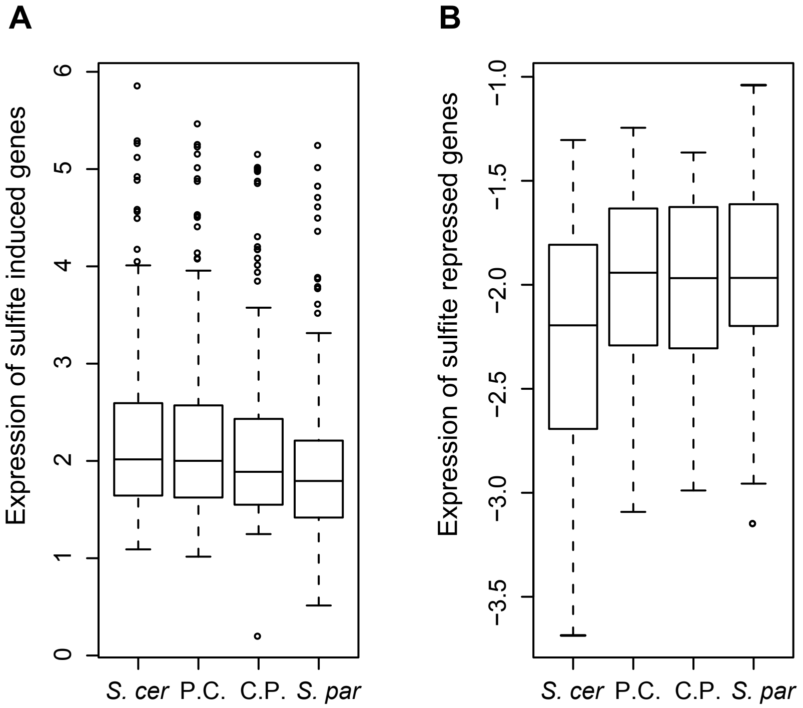

Figure S2: Sulfite-dependent changes in gene expression are larger for the *S. cerevisiae FZF1* allele relative to the *S. paradoxus* allele.

The log2 fold-change in expression as a result of sulfite treatment is shown for the *S. cerevisiae* (*S. cer*), *S. paradoxus* (*S. par*) and two 5' noncoding chimeric alleles of *FZF1* (C.P., P.C.). Box plots are shown for the 149 up-regulated genes (> 4-fold, $P < 0.01$) in panel A and 83 down-regulated genes (> 4-fold, $P < 0.01$) in panel B.
