# Supplementary figures and images for: Divergence of the Yeast Transcription Factor FZF1 Affects Sulfite Resistance
Source: PLoS Genet. 2012 Jun 14;8(6):e1002763. doi: 10.1371/journal.pgen.1002763 (PMC3375221; doi:10.1371/journal.pgen.1002763)

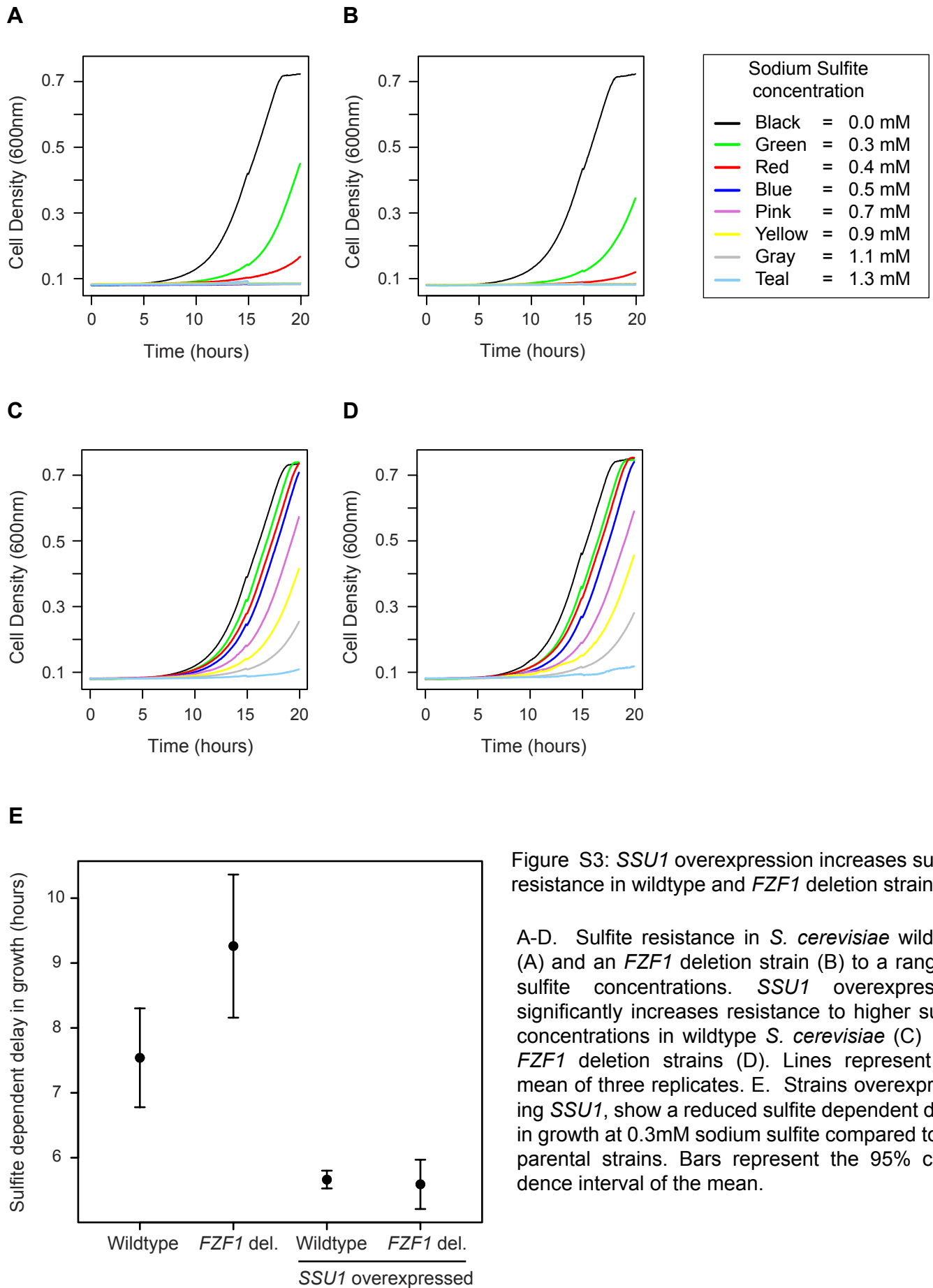

Supplement: Figure S3 — SSU1 overexpression increases sulfite resistance in wildtype and FZF1 deletion strains. A–D. Sulfite resistance in S. cerevisiae wildtype (A) and an FZF1 deletion strain (B) to a range of sulfite concentrations. SSU1 overexpression significantly increases resistance to higher sulfite concentrations in wildtype S. cerevisiae (C) and FZF1 deletion strains (D). Lines represent the mean of three replicates. E. Strains overexpressing SSU1, show a reduced sulfite dependent delay in growth at 0.3 mM sodium sulfite compared to the parental strains. Bars represent the 95% confidence interval of the mean. (PDF) [file pgen.1002763.s005.pdf]
