# Supplementary material for: Divergence of the Yeast Transcription Factor FZF1 Affects Sulfite Resistance
Source: PLoS Genet. 2012 Jun 14;8(6):e1002763. doi: 10.1371/journal.pgen.1002763 (PMC3375221; doi:10.1371/journal.pgen.1002763)
Supplement: Figure S4 — Variation in sulfite resistance within and between yeast species. Sulfite resistance is shown for 6 strains of S. cerevisiae, 5 strains of S. paradoxus, and a single strain of S. mikatae, S. castellii and K. lactis. Error bars show the 95% confidence interval of the mean. The error bars are within the circles for M8, M33 and YPS138. Sulfite resistance was also measured for a strain of S. kluyverii and S. bayanus, but the sulfite-dependent delay in growth could not be calculated since the strains grew in the presence of water (control) but not in the presence of sulfite. The wine strain M8 has the known translocation upstream of SSU1 that increases sulfite resistance [39], [57], [74]. (PDF) [file pgen.1002763.s006.pdf]

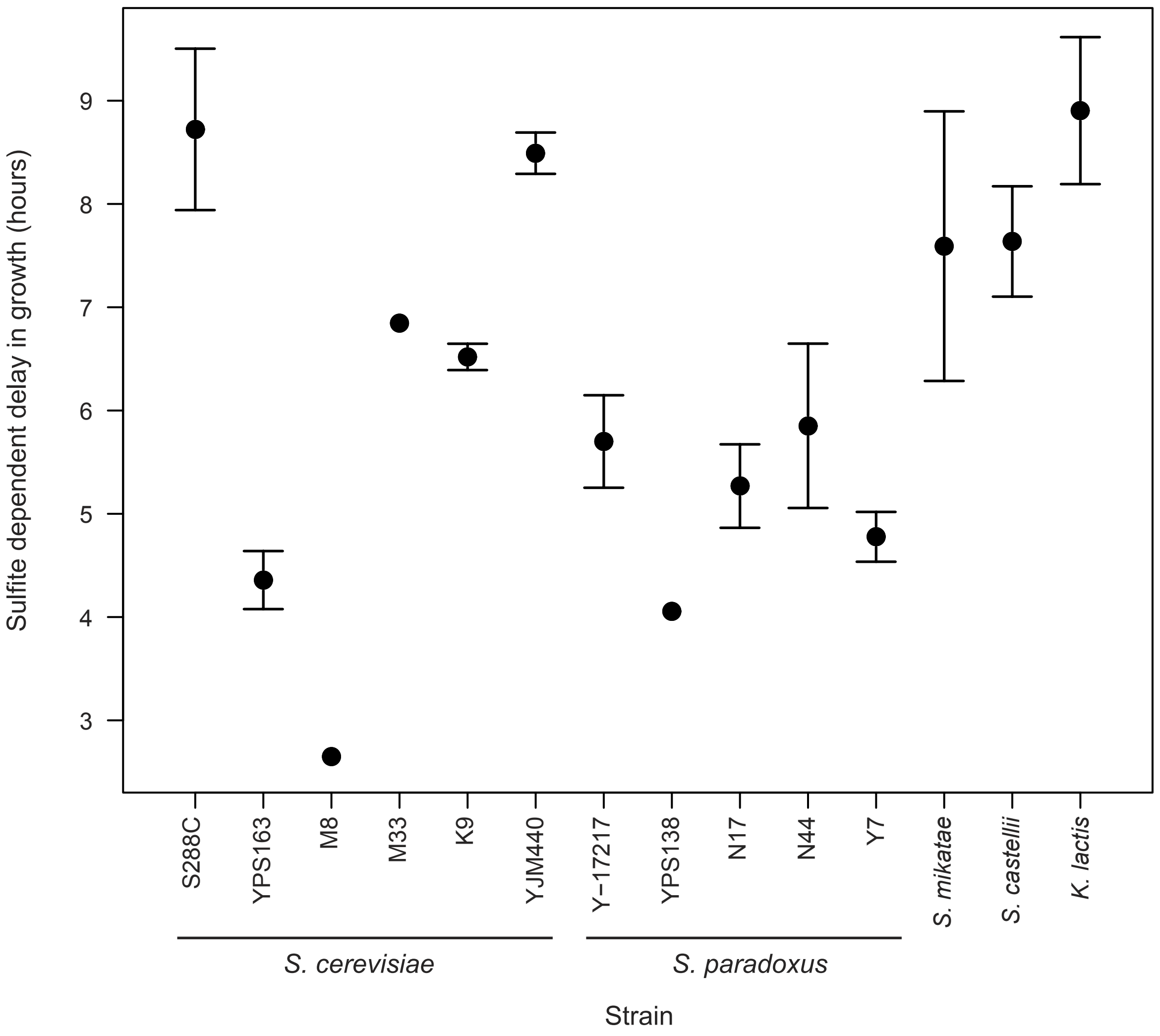

Figure S4: Variation in sulfite resistance within and between yeast species.

Sulfite resistance is shown for 6 strains of *S. cerevisiae*, 5 strains of *S. paradoxus*, and a single strain of *S. mikatae*, *S. castellii* and *K. lactis*. Error bars show the 95% confidence interval of the mean. The error bars are within the circles for M8, M33 and YPS138. Sulfite resistance was also measured for a strain of *S. kluyverii* and *S. bayanus*, but the sulfite-dependent delay in growth could not calculated since the strains grew in the presence of water (control) but not in the presence of sulfite. The wine strain M8 has the known translocation upstream of *SSU1* that increases sulfite resistance.
